# Impact of Prolonged Bisphosphonate Therapy on Atypical Femoral Fractures: Insights from a Single-Center Experience

**DOI:** 10.3390/jpm15120565

**Published:** 2025-11-21

**Authors:** Alessia Caldaci, Pierpaolo Panebianco, Sveva Condorelli, Noemy Zappalà, Luciano Costarella, Marco Sapienza, Gianluca Testa, Vito Pavone

**Affiliations:** Department of General Surgery and Medical Surgical Specialties, Section of Orthopedics and Traumatology, A.O.U. Policlinico-San Marco, University of Catania, Via Santa Sofia 78, 95123 Catania, Italy; alessia.c.92@hotmail.it (A.C.); Pierpaolopanebianco@gmail.com (P.P.); svevacondorelli@gmail.com (S.C.); noemyzappala@gmail.com (N.Z.); lcostarella@yahoo.it (L.C.); marcosapienza09@yahoo.it (M.S.)

**Keywords:** osteoporosis, bisphosphonates, atypical femoral fractures (AFF), clinical outcomes, radiographic outcomes

## Abstract

**Background:** Osteoporosis is a prevalent metabolic disorder frequently managed with bisphosphonates, which reduce fracture risk but, with prolonged use, may predispose to atypical femoral fractures (AFFs). These rare but serious complications highlight the need for individualized treatment strategies within the framework of precision medicine. **Methods:** We retrospectively analyzed six female patients (mean age around 70 years) with AFFs surgically treated at the University of Catania between September 2022 and March 2023. All patients had received bisphosphonate therapy for more than seven years. Demographic, clinical, and radiographic data were collected according to ASBMR criteria. Outcomes included fracture healing, hip and knee range of motion, quality of life (SF-36), and residual pain (VAS) at 12 months. **Results:** Fractures were subtrochanteric in two cases and mid-diaphyseal in four. All patients underwent intramedullary nailing. Mean radiographic union occurred at 5 ± 1.5 months, and functional recovery occurred at 4.5 ± 1.2 months. Quality of life declined from a pre-fracture SF-36 score of 77 to 57 at one year. Residual pain was minimal (VAS 2). **Conclusions:** Prolonged bisphosphonate therapy was strongly associated with AFFs, longer healing times, and reduced quality of life. These findings emphasize the importance of personalized management of osteoporosis, including risk-based treatment duration, tailored monitoring protocols, and early therapeutic adjustments to balance efficacy and safety in long-term care.

## 1. Introduction

Osteoporosis, affecting around 200 million women globally, is marked by reduced bone mass and deteriorated bone microarchitecture, leading to increased fracture risk. Predominantly seen in postmenopausal women due to estrogen decline, it also affects men and younger individuals with certain medical conditions or medications impacting bone health [1,2].

The primary treatment involves antiresorptive drugs like bisphosphonates (alendronate, risedronate, ibandronate, and zoledronic acid) and denosumab. These drugs inhibit osteoclast-mediated bone resorption, enhancing bone mineral density and reducing fracture risk. Denosumab, a monoclonal antibody, neutralizes RANKL, crucial for osteoclast formation and function.

Over the past two decades, there has been a notable increase in the incidence of osteoporosis and associated fragility fractures. This trend is largely attributed to the aging population, as the risk of osteoporosis and fractures increases with age. Concurrently, the use of bisphosphonates for both the prevention and treatment of osteoporosis has surged, making them the most prescribed medications for this condition.

Despite their proven efficacy in reducing the risk of vertebral, non-vertebral, and hip fractures, long-term bisphosphonate therapy has been associated with the occurrence of atypical femoral fractures (AFFs). AFFs are rare but serious complications that can occur with prolonged bisphosphonate use. These fractures typically occur in the subtrochanteric region or the diaphysis of the femur and are characterized by a transverse or short oblique configuration with minimal comminution.

The exact pathophysiological mechanisms underlying AFFs are not yet fully understood. However, current research suggests that prolonged bisphosphonate use leads to the inhibition of bone resorption and induces osteoclast apoptosis, resulting in the accumulation of microdamage and reduced bone remodeling. This, in turn, may contribute to the development of AFFs [3]. Although the absolute risk of AFFs remains low, these fractures have a significant negative impact on both short-term and long-term quality of life.

Patients with AFFs may experience loss of limb function, delayed healing, and increased mortality rates compared to those with typical femoral fractures. The clinical presentation of AFFs often includes prodromal symptoms such as thigh or groin pain, which can precede the fracture by weeks or months. Early recognition and intervention are crucial to prevent complete fractures and improve patient outcomes [4].

While bisphosphonates remain a cornerstone of osteoporosis therapy, emerging evidence indicates that the balance between benefits and risks varies according to treatment duration, comorbidities, and individual patient susceptibility [5]. These considerations underscore the importance of moving beyond a uniform approach and adopting personalized treatment strategies. In this context, integrating patient-specific risk profiles, early detection of prodromal symptoms, and stratified monitoring can help optimize therapy duration and reduce the likelihood of atypical complications.

The primary objective of this study is to evaluate the clinical and radiographic outcomes of patients with atypical bisphosphonate femoral fractures who were treated surgically in our Orthopedic Unit. By analyzing these outcomes, we aim to gain a better understanding of the impact of AFFs on patients’ quality of life and recovery process, and to identify potential strategies for improving the management and prevention of these fractures. Additionally, this study seeks to contribute to the growing body of literature on AFFs and provide insights into the long-term safety and efficacy of bisphosphonate therapy.

## 2. Materials and Methods

### 2.1. Declarations

This study was conducted in accordance with the Declaration of Helsinki and approved by the institutional review board of the University of Catania (approval code: Nr 25/2025/PO).

Informed consent was obtained from all patients for the publication of all their data and images. This comparative retrospective cohort study involved six female patients who underwent surgery for AFFs from bisphosphonates at the Department of Orthopedics and Traumatology of the University of Catania. We included all patients who underwent surgical treatment for femoral fractures classified as atypical, based on the criteria established by the American Society for Bone and Mineral Research (ASBMR).

### 2.2. Research Participants and Data Collection

These patients had a documented history of ongoing bisphosphonate therapy and were operated on between September 2022 and September 2023. To classify a fracture as atypical, we followed the ASBMR’s guidelines, which include both major and minor criteria [4]. For a fracture to be considered atypical, it had to meet at least four of the major criteria. These criteria include a transverse configuration at the lateral cortex of the femur, minimal or no comminution (fragmentation) of the fracture, the presence of a complete fracture or an incomplete fracture involving only the lateral cortex, periosteal or endosteal thickening at the fracture site, and the occurrence of the fracture with no or minimal trauma.

In addition to these major criteria, several minor criteria were considered, although they were not necessary for diagnosis. These minor criteria include generalized cortical thickening of the femoral shaft, bilateral fractures (fractures occurring in both femurs), a history of prodromal pain (pain preceding the fracture), and delayed healing of the fracture [4,5,6]. All patients were receiving pharmacological treatment with Alendronate.

The medical staff of the complex operative unit of Orthopedics of the A.O.U. Policlinico Rodolico-San Marco, University of Catania, examined various patient parameters, including gender, average age, the side of the femur affected (right or left), the specific location of the fracture (subtrochanteric or mid-diaphyseal), BMI, ASA score (American Society of Anesthesiologists), and time of surgery. (Table 1)

### 2.3. Surgical Treatment

All patients were treated surgically with a long intramedullary nail. The two patients with subtrochanteric fractures were also treated with a long intramedullary nail. All surgical procedures were performed by percutaneous procedures, without direct exposure to the fracture, using fluoroscopy for correct placement of the implant. No patient has developed post-operative infections. Following the diagnosis and surgical stabilization of the atypical femoral fracture, bisphosphonate therapy was discontinued for all patients in accordance with clinical guidelines. 

### 2.4. Outcomes

**Figure 1:**
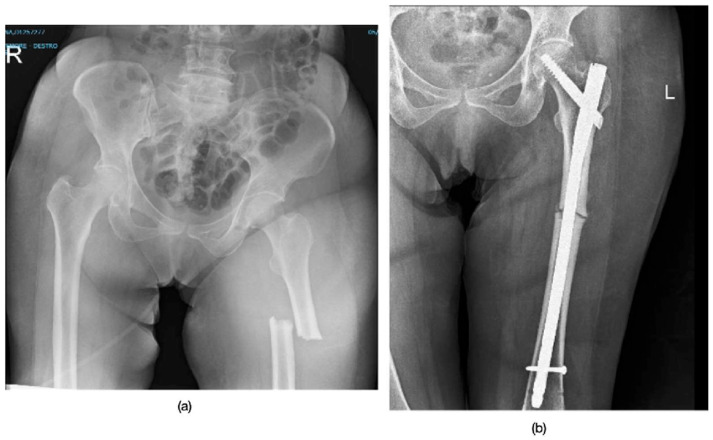
Radiographs of Patient 3, who sustained a subtrochanteric atypical femoral fracture. (**a**) The pre-operative X-ray explicitly shows the key diagnostic features of an AFF: the fracture originates from the lateral cortex, has a transverse configuration with minimal comminution, and displays localized periosteal thickening of the lateral cortex (beaking). (**b**) The post-operative image shows fracture stabilization with a long intramedullary nail.

Radiographic outcomes were assessed at 3, 6, 9, and 12 months post-surgery to monitor the healing process. Clinical outcomes were evaluated at 6 and 12 months post-surgery. The range of motion (ROM) of the knee and hip in flexion was also assessed during the immediate postoperative follow-up and at 6–12 months after surgery, to monitor recovery and evaluate the patient’s progress (Table 2 and Table 3, Figure 1).

To assess health-related quality of life, we administered the Short Form Health Survey (SF-36) questionnaire. This questionnaire consists of 36 items that measure both the physical and mental dimensions of the individual’s health, and it was used to compare the pre- and post-intervention states [7]. Residual pain at 12 months post-surgery was assessed using the pain visual analog scale (VAS), which provides a quantitative measure of pain intensity.

### 2.5. Statistical Analyses

Continuous data were expressed as mean ± standard deviation (SD). Due to the small sample size (*n* = 6), non-parametric tests were chosen for statistical comparisons. The Wilcoxon signed-rank test for paired data was used to compare pre- and post-operative SF-36 scores. A one-sample Wilcoxon signed-rank test was used to compare the mean healing times and VAS scores of our cohort against standard reference values. The threshold for statistical significance was set at *p* < 0.05. All analyses were performed using RStudio version 4.1.2 (RStudio Inc., Boston, MA, USA).

## 3. Results

### 3.1. Patient Demographics and Fracture Characteristics

The study involved six female patients, all around the age of 70. This is consistent with the higher incidence of osteoporosis in older women. The fractures were found in the right femur of four patients and the left femur of the remaining two. Specifically, two fractures were in the subtrochanteric region, while the other four were mid-diaphyseal. All fractures were complete and occurred on one side only.

### 3.2. Treatment and Healing Process

These patients had been on bisphosphonate therapy for an average of over seven years, which is significant since long-term use of these medications is linked to AFFs. Each fracture was surgically treated using a long intramedullary nail, a standard and effective method for such injuries. The table also indicates that no patient developed pseudoarthrosis (0%) (Table 4).

### 3.3. Quality of Life and Recovery

Before surgery (pre), 5 out of 6 of the patients reported a medium to high quality of life, with an SF-36 score of 77. However, a year after surgery (post), this percentage dropped to 70%, with an SF-36 score of 57, a statistically significant difference (*p* = 0.03). This decline indicates that the surgery and recovery period had a noticeable impact on their overall well-being.

Some patients even needed walking aids to improve their mobility; the clinical records noted that several patients required walking aids to improve their mobility, although the exact number of patients and duration of use were not systematically documented. The average time to regain hip and knee range of motion was 4.5 ± 1.2 months, which represents a significant delay compared to the 2–3 months typically required for functional recovery in standard femoral fractures (*p* = 0.04). On average, radiographic healing was achieved in 5 ± 1.5 months, a significantly longer period compared to the 3–4 months typically expected for standard femoral fractures (*p* = 0.03). Despite this, the residual pain reported after a year was very mild, with a VAS score of 2. Although this finding is statistically significant compared to a norm of zero pain (*p* < 0.0001), it represents a clinically minimal level of discomfort. (Table 5).

## 4. Discussion

Long-term use of bisphosphonates, a class of drugs commonly prescribed for the prevention and treatment of osteoporosis, is strongly associated with the development of AFFs [7,8,9].

In our study, we evaluated the impact of AFFs on patients’ quality of life using the SF-36 questionnaire. Quality of life is often overlooked in studies of atypical fractures, but it is essential to understand the real impact of these fractures on patients. Numerous studies support the association between prolonged bisphosphonate use and the development of atypical femoral fractures (AFF). For instance, Capeci et al. documented cases of bilateral low-energy femoral fractures in patients on long-term alendronate therapy [10]. Koh et al. discussed the challenges and controversies in the surgical management of AFFs related to bisphosphonate treatment [11].

The impact on quality of life, often overlooked, is crucial. Twelve months post-surgery, despite bone healing, a moderate reduction in quality of life is observed, aligning with other studies indicating long-term negative effects on patient functionality and well-being. Shkolnikova et al. highlighted how bisphosphonate-associated fractures significantly affect quality of life [12,13].

Our finding of prolonged healing times (five months for radiographic union and 4.5 months for functional recovery) is consistent with the hypothesis, supported by existing literature, that long-term bisphosphonate use alters bone metabolism. Studies suggest that the profound suppression of bone remodeling can interfere with the normal fracture healing process. While these drugs reduce osteoporotic fracture risk, they can interfere with normal bone remodeling and slow fracture healing. Komatsubara et al. observed microdamage accumulation in bone tissue following long-term bisphosphonate treatment, suggesting a mechanism for prolonged healing times [14].

Mashiba et al. noted that bisphosphonate-induced suppression of bone turnover increases microdamage accumulation and reduces certain biomechanical properties of bone [15]. Additionally, Park et al. reported a case of sequential subtrochanteric femoral fracture following an atypical diaphyseal fracture in a long-term bisphosphonate-treated patient, underscoring these patients’ vulnerability to further fractures [16].

Bisphosphonates, including alendronate, risedronate, ibandronate, and zoledronic acid, inhibit osteoclast-mediated bone resorption, effectively reducing typical fracture risk. However, prolonged use may increase susceptibility to atypical femoral fractures (AFFs). Studies indicate that extended suppression of bone remodeling by bisphosphonates can lead to microdamage accumulation, weakening bone and increasing fracture risk, even with minimal trauma [11,12,13,14,15,16].

The study found prolonged healing times, with an average of 5 months for radiographic union and 4.5 months for recovery of range of motion (ROM). This finding is notable as a clear consensus on healing times for AFFs is lacking in the literature; for instance, studies by Weil et al. [17] report variable outcomes, while others by Koh et al. and Savaridas et al. do not specify precise timelines [11,18]. While this delay—likely due to altered bone metabolism from bisphosphonates—did not result in pseudoarthrosis, it was associated with persistent mild pain (VAS 2 at 12 months) and a 20% reduction in quality of life (SF-36). The need for walking aids further underscores patients’ difficulty in achieving complete functional recovery (Table 6).

Our study found an average radiographic healing time of five months. Although we did not have a direct control group, this is longer than the three to four months typically reported in the literature for standard osteoporotic fractures treated with the same surgical method [13,14,15,16,17,18]. This finding supports the hypothesis that the altered bone remodeling caused by long-term bisphosphonate use contributes to a delay in the healing of atypical fractures.

As observed, there is no unanimous consensus on the healing times of AFF in the literature. Some studies, like that of Weil et al., do not report specific healing times, while others, such as Koh et al. and Savaridas et al., focus on other aspects of treatment and prognosis of AFF [11,17,18].

In diagnosis, the use of known diagnostic criteria is essential; the most important are those established by the ASBMR, which include distinctive radiographic features such as fracture origin from the lateral cortex, transverse course, poor comminution, and periosteal thickening [19]. Capeci et al., Goh et al., and Shkolnikova et al. highlight specific radiographic configurations that can also be seen in our study, namely lateral cortical thickening, transverse fracture, and medial cortical beak [10,13,20].

The combination of these radiographic features is highly suggestive of AFFs. Recognition of these signs is essential for early diagnosis and appropriate treatment. Early diagnosis of AFFs is critical to prevent progression to a complete fracture and to reduce the risk of complications. It is important to carefully evaluate radiographs to detect signs of stress or incomplete fracture in the contralateral femur. Bilaterality is a common feature in AFFs, and early identification of radiographic changes in the other femur may allow for preventive measures to be taken to reduce the risk of contralateral fractures.

A key concept that emerges from several studies is that the accumulation of microdamage at the bone level is caused by the prolonged use of bisphosphonates, which, by inhibiting the activity of osteoclasts, slow down the process of resorption and remodeling of bone tissue [12,13,14]. An important aspect in the inhibition of remodeling is that this mechanism can lead to an increase in bone mineralization, making the bone more rigid and less resistant to stress [11,12,14,15].

There are other factors, such as genetic predisposition, advanced age, and the presence of other pathologies, which could contribute to the development of these fractures [11,12,13]. Although the causal relationship between the prolonged use of bisphosphonates and AFFs is still not well defined, our study, like others in the literature, highlights the importance of an accurate risk-benefit assessment before starting long-term therapy with bisphosphonates, and of careful monitoring of patients during treatment.

For the treatment of AFFs, withdrawal of bisphosphonate therapy is generally recommended, although the impact of this measure on fracture healing is still debated [11,12,13,14,15,16]. Surgical treatment, particularly intramedullary nailing, is considered the gold standard for complete fractures.

However, the literature shows a high incidence of postoperative complications in AFFs, including non-union, which often requires revision surgery. Koh et al. showed that intramedullary nailing was the predominant treatment choice for complete AFFs, noting that intramedullary nailing was preferred over plate fixation due to its better load-sharing ability and less flexural motion across the fracture site [11].

Savaridas et al. conducted a study in rat models of direct fracture healing and reported that bisphosphonates had an inhibitory effect on direct healing [18].

Weil et al. described a series of 15 patients with 17 AFFs associated with long-term bisphosphonate use. Seven of these fractures were treated with intramedullary nailing but required revision surgery. These authors attributed the high failure rate to impaired bone healing due to prolonged bisphosphonate therapy. However, the study did not find that healing time was different in those who had been treated with bisphosphonates for more than 5 years compared with those who had been treated for less than 5 years [17].

Therefore, Weil et al. recommend that the choice between nailing and plate fixation for AFFs should be guided by the specific characteristics of the patient and the fracture [17]. This matches the findings of our study and the conclusions of many other studies emphasizing the need for an individualized approach in the management of AFFs. Some studies in the literature have examined the efficacy of teriparatide (TPTD) in treating fractures in these patients. Previous results suggest that TPTD could promote better healing of fractures by reducing delayed and non-union rates and shortening overall recovery times.

In their meta-analysis, Byun et al. examined the effect of treatment with TPTD on AFFs, comparing delayed union, non-union, and fracture healing rates between patients treated or not treated with TPTD. The results showed that patients who did not receive TPTD had a higher incidence of delayed union and non-union, as well as longer healing times than those treated with TPTD [21].

Kendler et al. compared the effectiveness of TPTD with risedronate in preventing new fractures in patients with severe osteoporosis. In their randomized, double-blind clinical trial, postmenopausal women with vertebral fractures and low bone mineral density were assigned to receive TPTD or risedronate for 24 months. The results revealed that the TPTD group had a significantly lower incidence of new vertebral fractures than the risedronate group. The number of clinical and non-vertebral fractures was also lower in the TPTD group, indicating that TPTD is more effective than risedronate [22]. The management of incomplete fractures, particularly those associated with long-term bisphosphonate use, presents a significant challenge. The presence or absence of a radiolucent line in the incomplete fracture is a critical factor in treatment decision-making.

The radiolucent line is a radiographic sign that indicates a high likelihood of progression to a complete fracture. This distinguishing feature helps distinguish between stable incomplete fractures that can be managed conservatively and those at risk of complete fracture that may require prophylactic surgery. Koh et al. demonstrated that many patients with a radiographically visible radiolucent line had a complete fracture, whereas those without such a sign had uneventful healing with a conservative approach [11].

This observation is also supported by research by Saleh et al., who found a clear difference in the clinical outcome of incomplete fractures based on the presence or absence of the radiolucent line. Patients without the line showed complete healing with conservative treatment, while those with the line had a high likelihood of progression to a complete fracture, often requiring surgery [22,23,24]. Our study, in line with the literature, highlights the importance of the radiolucent line as a decision factor in the treatment of incomplete femoral fractures associated with the use of bisphosphonates.

The presence of this line, visible on radiography, indicates a significantly higher risk of progression to a complete fracture, requiring a different management approach compared to incomplete fractures without this sign.

All patients were treated for complete fractures. However, a review of prior radiographs revealed that three of the six patients (50%) had evidence of an incomplete stress fracture with a visible radiolucent line before it progressed to a complete fracture. All fractures occurred on one side only.

This finding is significant because patients with this feature showed a worrying trend: a high incidence of progression to a complete fracture, despite a period of conservative treatment. This highlights the fragility of these fractures and the insufficiency of the conservative approach alone in many cases. The present study also found a considerable mean duration of treatment with bisphosphonates (7.3 years). This result is important because literature suggests an increased risk of atypical fractures with prolonged use of bisphosphonates, particularly after 5 years. The analysis of the duration of treatment supports a possible correlation between the prolonged use of bisphosphonates and the onset of these fractures. By using the ASBMR criteria for the diagnosis of atypical fractures, we aimed to achieve greater accuracy in the classification of cases.

These criteria, which are widely accepted by the scientific community, allow us to distinguish atypical fractures from typical fractures, thus contributing to the validity of the study.

When interpreting our findings—such as the prolonged healing times and impact on quality of life—it is crucial to consider the context of our study’s limitations. With only six patients, it is not possible to determine the specific influence of individual clinical variables like BMI, comorbidities (as indicated by ASA scores), or previous fracture history on the outcomes. Therefore, our results should be viewed as a descriptive overview of the clinical journey in this small cohort, rather than a definitive analysis of prognostic factors. Future, larger studies are needed to parse the effects of these co-factors from the effects of the AFF itself.

The study has some important limitations to consider. The primary and most significant limitation of this study is its very small sample size. With only six patients, the statistical power of our analyses is low, which severely restricts the generalizability of our findings to a broader population. Therefore, the conclusions presented here should be considered preliminary and require validation through larger, prospective, multi-center studies. Furthermore, the absence of a contemporaneous control group of patients with typical osteoporotic fractures prevents a direct comparison of healing outcomes within our institution. The retrospective design introduces a potential for selection bias and inconsistent data recording, which may have influenced the outcomes.

Beyond the general association between prolonged bisphosphonate therapy and AFFs, our findings underscore the need for a personalized approach in osteoporosis management. Specifically, the average treatment duration of over seven years in our cohort reinforces the importance of tailoring therapy length to the individual patient’s risk profile. Monitoring strategies such as periodic imaging for early signs of incomplete fractures, careful evaluation of prodromal pain, and stratification by clinical risk factors (age, BMI, comorbidities, previous fractures) may allow for earlier detection and intervention. Personalized treatment adjustments—such as considering a “drug holiday,” switching to anabolic agents like teriparatide in selected patients, or intensifying monitoring in those at higher risk—could minimize complications while preserving anti-fracture benefits. Thus, our study supports the integration of risk-based monitoring and individualized treatment duration into clinical decision-making for patients on long-term bisphosphonates.

## 5. Conclusions

This study highlights a strong correlation between prolonged bisphosphonate use and the development of atypical femoral fractures (AFFs). It emphasizes the need for careful risk-benefit assessment before initiating long-term therapies.

Atypical fractures exhibit longer healing times and negatively impact post-surgery quality of life. Radiolucent lines are identified as a key prognostic factor, and the ASBMR criteria improve diagnostic accuracy. This study calls for further research to improve treatment and prevention strategies.

In line with the principles of precision medicine, our results suggest that prolonged bisphosphonate therapy should not follow a “one-size-fits-all” model. Instead, treatment duration, monitoring frequency, and therapeutic adjustments should be individualized according to patient-specific factors, including fracture risk, comorbidities, and early radiographic changes. Personalized strategies—such as identifying those most likely to benefit from continued therapy versus those who may require alternative treatment or earlier intervention—represent a crucial step toward improving outcomes and reducing the burden of atypical fractures in osteoporosis care.

## Figures and Tables

**Table 1:** Demographic data.

	SEX	BMI	Fracture Type	Side Fracture	Time of Surgery	ASA Score
*Patient 1*	F	20	Subtrochanteric	Left	48 h	2
*Patient 2*	F	23	Mid-third diaphysis	Right	48 h	2
*Patient 3*	F	31	Subtrochanteric	Left	24 h	2
*Patient 4*	F	22	Mid-third diaphysis	Right	48 h	3
*Patient 5*	F	26	Mid-third diaphysis	Right	72 h	2
*Patient 6*	F	18.5	Mid-third diaphysis	Right	48 h	3

**Table 2:** Knee ROM in flexion follow-up.

	Post-Surgery	6 Months	12 Months
*Patient 1*	0–40°	0–110°	0–130°
*Patient 2*	0–30°	0–109°	0–120°
*Patient 3*	0–40°	0–120°	0–140°
*Patient 4*	0–40°	0–110°	0–140°
*Patient 5*	0–20°	0–100°	0–110°
*Patient 6*	0–40°	0–110°	0–130°

**Table 3:** Hip ROM in flexion follow-up.

	Post-Surgery	6 Months	12 Months
*Patient 1*	0–30°	0–80°	0–120°
*Patient 2*	0–20°	0–80°	0–110°
*Patient 3*	0–40°	0–90°	0–140°
*Patient 4*	0–30°	0–80°	0–110°
*Patient 5*	0–20°	0–90°	0–110°
*Patient 6*	0–30°	0–85°	0–110°

**Table 4:** Patient and fracture characteristics.

Mean Age	Involved Side	Localization	Time of Use of Bisphosphonates	Surgical Treatment	Pseudoarthrosis
70.3 ± 6.5 y	4 Right, 2 Left	2 subtrochanteric 4 mid-diaphyseal	7.3 ± 1.5 y	long intramedullary nail	0%

**Table 5:** Bone healing, quality of life, and residual pain after surgery.

Radiographic Healing Time	Quality of Life	ROM Average Recovery Time	Residual Pain at 12 Months
5 ± 1.5 months	SF36 of 77 ± 15.4 pre (before surgery) SF36 of 57 ± 12.5 post (a year after surgery)	4.5 ± 1.2 months	VAS of 2 ± 1

**Table 6:** Comparative table of healing time.

Study	Radiographic Healing	Recovery of ROM
Our Study	5 months	4.5 months
Weil et al. [17]	Variable	Variable
Koh et al. [11]	Not specific	Not specific
Savaridas et al. [18]	Not specific	Not specific

## Data Availability

The original contributions of this study are included in the article. Further inquiries can be directed to the corresponding authors.

## References

[1] Khandelwal S., Lane N.E. (2023). Osteoporosis: Review of Etiology, Mechanisms, and Approach to Management in the Aging Population. Endocrinol. Metab. Clin. N. Am..

[2] Johnston C.B., Dagar M. (2020). Osteoporosis in Older Adults. Med. Clin. N. Am..

[3] Ural A. (2021). Biomechanical mechanisms of atypical femoral fracture. J. Mech. Behav. Biomed. Mater..

[4] Ebrahimpour A., Sadighi M., Hoveidaei A.H., Chehrassan M., Minaei R., Vahedi H., Mortazavi S.J. (2021). Surgical Treatment for Bisphosphonate-related Atypical Femoral Fracture: A Systematic Review. Arch. Bone Jt. Surg..

[5] Farrukh A.M., Reyes L.C.F., Capa G.S.L., Padilla T.B.M., Sunkara V., Dhakal S. (2023). Bisphosphonate-induced atypical femoral shaft fracture: A case report. Radiol. Case Rep..

[6] LeBlanc E.S., Rosales A.G., Genant H.K., Dell R.M., Friess D.M., Boardman D.L., Santora A.C., Bauer D.C., de Papp A.E., Black D.M. (2019). Radiological criteria for atypical features of femur fractures: What we can learn when applied in a clinical study setting. Osteoporos. Int..

[7] Lins L., Carvalho F.M. (2016). SF-36 total score as a single measure of health-related quality of life: Scoping review. SAGE Open Med..

[8] Toro G., Braile A., Liguori S., Moretti A., Landi G., Cecere A.B., Conza G., De Cicco A., Tarantino U., Iolascon G. (2023). The role of the fracture liaison service in the prevention of atypical femoral fractures. Ther. Adv. Musculoskelet. Dis..

[9] Çakmak S., Mahiroğulları M., Keklikçi K., Sarı E., Erdik B., Rodop O. (2013). Bilateral low-energy sequential femoral shaft fractures in patients on long-term bisphosphonate therapy. Acta Orthop. Traumatol. Turc..

[10] Capeci C.M., Tejwani N.C. (2009). Bilateral low-energy simultaneous or sequential femoral fractures in patients on long-term alendronate therapy. J. Bone Jt. Surg. Am..

[11] Koh A., Guerado E., Giannoudis P.V. (2017). Atypical femoral fractures related to bisphosphonate treatment: Issues and controversies related to their surgical management. Bone Jt. J..

[12] Kwek E.B., Goh S.K., Koh J.S., Png M.A., Howe T.S. (2008). An emerging pattern of subtrochanteric stress fractures: A long-term complication of alendronate therapy?. Injury.

[13] Shkolnikova J., Flynn J., Choong P. (2013). Burden of bisphosphonate-associated femoral fractures. ANZ J. Surg..

[14] Komatsubara S., Mori S., Mashiba T., Ito M., Li J., Kaji Y., Akiyama T., Miyamoto K., Cao Y., Kawanishi J. (2003). Long-term treatment of incadronate disodium accumulates microdamage but improves the trabecular bone microarchitecture in dog vertebra. J. Bone Miner. Res..

[15] Mashiba T., Saito M., Yamagami Y., Tanaka M., Iwata K., Yamamoto T. (2017). Effects of suppressed bone remodeling by minodronic acid and alendronate on bone mass, microdamage accumulation, collagen crosslinks and bone mechanical properties in the lumbar vertebra of ovariectomized cynomolgus monkeys. Bone.

[16] Park K.T., Lee K.B. (2015). Sequential subtrochanteric femoral fracture after atypical diaphyseal fracture in a long-term bisphosphonate user: A case report. Acta Chir. Orthop. Traumatol. Cech..

[17] Weil Y.A., Rivkin G., Safran O., Liebergall M., Foldes A.J. (2011). The outcome of surgically treated femur fractures associated with long-term bisphosphonate use. J. Trauma..

[18] Savaridas T., Wallace R.J., Salter D.M., Simpson A.H. (2013). Do bisphosphonates inhibit direct fracture healing: A laboratory investigation using an animal model. Bone Jt. J..

[19] Phillips H.K., Harrison S.J., Akrawi H., Sidhom S.A. (2017). Retrospective review of patients with atypical bisphosphonate related proximal femoral fractures. Injury.

[20] Goh S.K., Yang K.Y., Koh J.S., Wong M.K., Chua S.Y., Chua D.T., Howe T.S. (2007). Subtrochanteric insufficiency fractures in patients on alendronate therapy: A caution. J. Bone Jt. Surg. Br..

[21] Byun S.E., Lee K.J., Shin W.C., Moon N.H., Kim C.H. (2023). The effect of teriparatide on fracture healing after atypical femoral fracture: A systematic review and meta-analysis. Osteoporos. Int..

[22] Kendler D.L., Marin F., Zerbini C.A.F., Russo L.A., Greenspan S.L., Zikan V., Bagur A., Malouf-Sierra J., Lakatos P., Fahrleitner-Pammer A. (2018). Effects of teriparatide and risedronate on new fractures in post-menopausal women with severe osteoporosis (VERO): A multicentre, double-blind, double-dummy, randomised controlled trial. Lancet.

[23] Saleh A., Hegde V.V., Potty A.G., Schneider R., Cornell C.N., Lane J.M. (2012). Management strategy for symptomatic bisphosphonate-associated incomplete atypical femoral fractures. HSS J..

[24] Mangano G.R.A., Avola M., Blatti C., Caldaci A., Sapienza M., Chiaramonte R., Vecchio M., Pavone V., Testa G. (2022). Non- Adherence to Anti-Osteoporosis Medication: Factors Influencing and Strategies to Overcome It. A Narrative Review. J. Clin. Med..

